# Retraction Note to: Degradation of long non-coding RNA-CIR decelerates proliferation, invasion and migration, but promotes apoptosis of osteosarcoma cells

**DOI:** 10.1186/s12935-022-02465-6

**Published:** 2022-01-24

**Authors:** Shiwei Liu, Jingchao Li, Liang Kang, Yueyang Tian, Yuan Xue

**Affiliations:** 1 Department of Orthopaedics, Tianjin Medical University General Hospital, 154 Anshan Road, Heping District, Tianjin, 300052 China; 2 Tianjin Key Laboratory of Spine and Spinal Cord, Tianjin Medical University General Hospital, 154 Anshan Road, Heping District, Tianjin, 300052 China

## Retraction to: Cancer Cell Int (2019) 19:349 10.1186/s12935-019-1076-7

The Editors-in-Chief have retracted this article. After publication, concerns were raised about the images presented in Figs. 2–8. Specifically:

- Repeated motifs are present in colony formation images in Figs. 2a and 3a;
- Repeated scatter patterns of data points appear in Figs. 4b and 5b;
- Cell images in various panels of Figs. 6a and 7a partially overlap;
- Tumor images appear to be presented on an artificial background in Figs. 8a and 8c.
- The corresponding author has stated the original data is no longer available. The Editor-in-Chief therefore no longer has confidence in the presented data.

All authors agree to this retraction.

